# Pediatric autoimmune diseases in the light of COVID-19 pandemic, A retrospective observational big data study

**DOI:** 10.1016/j.jtauto.2025.100281

**Published:** 2025-03-06

**Authors:** Rim Kasem Ali Sliman, Hilla Cohen, Shereen Shehadeh, Reut Batcir, Yigal Elenberg Alter, Keren Cohen, Ilana Koren, Inbal Halabi, Hussein Sliman, Mohamad Hamad Saied

**Affiliations:** a Technion Israel Institute of Technology, Rappaport Faculty of Medicine, Haifa 3109601, Israel; b Department of Pediatrics, Clalit Health Care Organization, Carmel Medical Center, Haifa, Israel; c Clalit Health Care Organization, Carmel Medical Center, Haifa, Israel; d Infectious Disease Unit, Carmel Medical Center, Haifa, Israel; e Pediatric Gastroenterology Unit, Carmel Medical Center, Haifa, Israel; f Pediatric Endocrine Unit, Carmel Medical Center, Haifa, Israel; g Department of Cardiology, Carmel Medical Center, Heart Center, Haifa, Israel; h Department of Pediatric Immunology and Infectious Diseases, Wilhelmina Children's Hospital/University Medical Center, Utrecht, the Netherlands

**Keywords:** COVID-19, SARS-CoV-2, Autoimmune diseases, Children, Incidence rate, Epidemiology

## Abstract

**Background:**

The COVID-19 pandemic has raised concerns about potential links between SARS-CoV-2 infection and autoimmune diseases. This study investigated changes in the incidence rate (IR) of autoimmune diseases among children following the pandemic's onset.

**Methods:**

A retrospective cross-sectional study analyzed data from Clalit Health Services, Israel's largest healthcare provider, examining the IR of different autoimmune diseases in children aged 0–18. The study compared pre-pandemic (2019) with pandemic/post-pandemic periods (2020–2023), encompassing a cohort of over 1.5 million children.

**Results:**

Significant IR increases were observed across multiple autoimmune diseases. Rheumatic diseases (Juvenile Idiopathic Arthritis, Systemic Lupus Erythematosus, Henoch Schoenlein Purpura (HSP)) showed consistent increases, with HSP demonstrating the most pronounced trend. Endocrine disorders exhibited diverse patterns, with autoimmune thyroid diseases and Type 1 diabetes showing overall increases, while diabetic ketoacidosis exhibited an initial spike followed by a decline. Gastrointestinal diseases displayed heterogeneous patterns; Celiac disease and Ulcerative colitis showed general increases, Crohn's disease showed a downward trend, and autoimmune hepatitis exhibited an initial significant decrease followed by a significant increase. Dermatological conditions, including Psoriasis and Vitiligo, demonstrated consistent elevations throughout 2020–2023. Immune Thrombocytopenia Purpura showed initial decreases followed by significant increases in 2022–2023.

**Conclusions:**

This comprehensive analysis reveals significant changes in pediatric autoimmune disease incidence following the COVID-19 pandemic, suggesting potential associations between SARS-CoV-2 infection and autoimmune dysregulation. The diverse patterns observed across different conditions highlight the complex interplay between viral infection and autoimmunity, emphasizing the need for continued surveillance and investigation of long-term immunological consequences of COVID-19 in pediatric populations.

## Abbreviations

ACE2: Angiotensin-Converting Enzyme 2
ADEM: Acute Disseminated Encephalomyelitis
AIH: Autoimmune Hepatitis
AITD: Autoimmune Thyroid Disease
CD: Celiac Disease
CHS: Clalit Health Services
CMV: Cytomegalovirus
DKA: Diabetic Ketoacidosis
DNA: Deoxyribonucleic Acid
EBV: Epstein-Barr Virus
ESPGHAN: European Society for Pediatric Gastroenterology, Hepatology and Nutrition
GD: Graves' Disease
HbA1c: Glycated Hemoglobin
HLH: Hemophagocytic Lymphohistiocytosis
HSP: Henoch-Schönlein Purpura
HTLV-1: Human T-Lymphotropic Virus Type 1
IBD: Inflammatory Bowel Disease
IL-6: Interleukin 6
IR: Incidence Rate
IRB: Institutional Review Board
ITP: Immune Thrombocytopenic Purpura
JIA: Juvenile Idiopathic Arthritis
KD: Kawasaki Disease
MIS: Multisystem Inflammatory Syndrome
MS: Multiple Sclerosis
NET: Neutrophil Extracellular Trap
PBC: Primary Biliary Cholangitis
PICU: Pediatric Intensive Care Unit
RA: Rheumatoid Arthritis
SARS-CoV-2: Severe Acute Respiratory Syndrome Coronavirus 2
SLE: Systemic Lupus Erythematosus
T1D: Type 1 Diabetes
UC: Ulcerative Colitis

## 1 Background

Autoimmune diseases encompass a diverse spectrum of disorders in which immune system dysregulation triggers responses against self-antigens, leading to tissue damage and organ dysfunction. Though the exact etiology remains unknown, research has revealed that various factors are believed to contribute to their development, including genetic predisposition, gut microbiome alterations, various environmental triggers, and infectious agents such as bacterial, viral, fungal, and parasitic infections, along with physical and hormonal factors [[1], [2], [3], [4], [5], [6], [7]].

The relationship between viral infections and autoimmunity has been extensively documented across a spectrum of conditions, including Systemic Lupus Erythematosus (SLE), Rheumatoid Arthritis (RA), Sjogren's syndrome, systemic vasculitis, Celiac Disease (CD), Multiple Sclerosis (MS), Primary Biliary Cholangitis (PBC), polymyositis, uveitis, Henoch-Schönlein Purpura (HSP), Systemic Juvenile Idiopathic arthritis (JIA), systemic sclerosis, Hashimoto thyroiditis and Autoimmune Hepatitis (AIH) [[8], [9], [10], [11], [12], [13], [14],^18^]. The most prominent pathogenic viruses that have been proposed in the triggering and initiation of autoimmune diseases include Parvovirus B19, Epstein-Barr-virus (EBV), Cytomegalovirus (CMV), Herpes virus-6, HTLV-1, Hepatitis A and C virus, and Rubella virus [11,[15], [16], [17], [18], [19], [20], [21]].

Viral infections can initiate autoimmune pathogenesis through diverse molecular and cellular mechanisms, which can operate independently or synergistically. Bystander activation represents a crucial mechanism wherein viral infection induces a pro-inflammatory microenvironment that leads to non-specific activation of autoreactive T cells [22,23]. This process involves the release of cytokines, chemokines, and co-stimulatory molecules that can break peripheral tolerance and activate previously dormant autoreactive lymphocytes. Studies have demonstrated that type I interferons produced during viral infections can serve as a critical mediator in this process, promoting the expansion and activation of autoreactive T cells that would typically remain suppressed [24,25].

Molecular mimicry represents another fundamental mechanism where viral antigens share structural similarities with host proteins, leading to cross-reactive immune responses [25,26]. This phenomenon is exemplified by EBV infection, where viral EBNA-1 protein shares sequence similarity with lupus autoantigens, potentially contributing to SLE development [23]. Similarly, CMV peptides demonstrate structural homology with myelin basic protein, possibly contributing to Multiple Sclerosis pathogenesis [12,17,22,23,[25], [26], [27]]. Epitope spreading further contributes significantly to autoimmune progression through the exposure of previously sequestered self-antigens following initial tissue damage [22]. This process involves the diversification of epitopes recognized by T and B cells, expanding from the initial focused response to encompass other epitopes on the same or different proteins. In type 1 diabetes, viral-induced damage to pancreatic β-cells can expose multiple islet antigens, leading to a broader autoimmune response [20].

NET (Neutrophil Extracellular Trap) formation represents another crucial mechanism in autoimmunity development [28]. While primarily serving as antimicrobial defenses, NETs can become sources of autoantigens, particularly in conditions like SLE and vasculitis. Studies have demonstrated that unregulated NET formation during viral infections exposes nuclear and cytoplasmic autoantigens, promoting anti-nuclear antibody development [24,28]. Furthermore, persistent viral persistence and chronic infection maintain a persistent inflammatory state, leading to continuous immune activation and potential breakdown of self-tolerance [25]. The ensuing direct tissue damage and inflammation trigger the release of damage-associated molecular patterns and altered self-antigens, activating pattern recognition receptors and subsequent inflammatory cascade [[29], [30], [31]]. This process is further complicated by viral-mediated disruption of regulatory T cell (Treg) function, a crucial component of immune tolerance maintenance [[32], [33], [34], [35]]. The interplay between these mechanisms creates a self-perpetuating cycle that can initiate and sustain autoimmune responses.

In December 2019, a novel outbreak of a new strain of coronavirus infection emerged, the SARS-CoV-2 or COVID-19, subsequently designated a global pandemic by March 2020 [1]. COVID-19 demonstrated varying clinical presentation severity across age groups, with children generally exhibiting milder manifestations than adults [36]. Apart from the acute severe infection, several complications, including autoimmune diseases, have been noted [10,[37], [38], [39]]. The shared pathogenetic mechanisms and clinical-radiological features between COVID-19 and hyper-inflammatory diseases suggest that SARS-CoV-2 may trigger autoimmune and autoinflammatory dysregulation through multiple pathways [1,40]. Several studies have demonstrated a range of autoantibodies in patients with COVID-19, including antinuclear antibodies (35.6 %), anti-Ro/SSA (25 %), rheumatoid factor (19 %), lupus anticoagulant (11 %), and antibodies against interferon-I (10 %) [10]. Sequence analysis revealed 28 human proteins containing regions homologous to SARS-CoV-2 peptides, with these proteins being targets of autoantibodies present in various established autoimmune disorders [10,41,42].

The clinical spectrum of COVID-19-associated autoimmune manifestations ranges from organ-specific conditions such as cutaneous vasculitis, immune thrombocytopenic purpura (ITP), transverse myelitis, Guillain–Barré syndrome, as well as systemic autoimmune and inflammatory disorders including systemic vasculitis, hemophagocytic lymphohistiocytosis (HLH), SLE, Kawasaki disease (KD), autoimmune hemolytic anemia, Graves' disease (GD), Hashimoto's thyroiditis and neurologic demyelinating syndromes such as Acute Disseminated Encephalomyelitis (ADEM) [10,[43], [44], [45], [46], [47], [48], [49], [50], [51], [52]].

Our study aims to investigate potential changes in the incidence rate (IR) of various autoimmune diseases following the onset of the COVID-19 pandemic.

## 2 Methods

Clalit Health Services (CHS) is an Israeli payer-provider integrated healthcare system, serving more than 4.5 million members, which includes 54 % of the Israeli population. It collects data from its different operational systems (hospitals, primary clinics, specialty clinics, pharmacies, laboratories, diagnostic and imaging centers, allied health services, dental and complementary health services, as well as organizational and national disease registries) into a unified analytical data warehouse that is used for research. Thus, the “Research Data Center” is authorized to access and extract data from the warehouse for research purposes. Extraction was automatic after the study personnel defined the required fields.

Our study is a retrospective cross-sectional study. It was approved by the Institutional Review Board (IRB) and done by the Declaration of Helsinki and good clinical practice guidelines number 0017-23-CMC**.** All children (aged 0–18 years) insured by the CHS in Israel between January 1, 2019 and December 31, 2023 were included. With a new diagnosis in the medical file of one of the listed common autoimmune diseases:

• Rheumatological Conditions
  o JIA
  o SLE
  o HSP
• Endocrine Disorders
  o Autoimmune Thyroid Disease (AITD)
    ⁃ Hypothyroidism (including Hashimoto Thyroiditis)
    ⁃ GD
  o Type 1 Diabetes (T1D) and associated diabetic ketoacidosis (DKA)
• Gastrointestinal Conditions
  o AIH
  o CD
  o Inflammatory Bowel Disease (IBD)
    ⁃ Crohn's Disease
    ⁃ Ulcerative Colitis (UC)
• Hematologic Conditions
  o ITP
• Dermatologic Conditions
  o Psoriasis
  o Vitiligo

## 3 Study design and statistics

Statistical analyses were conducted using R statistical software (Version 4.2.0; R Foundation for Statistical Computing, Vienna, Austria) in RStudio (Version 2022.02.3 Build 492, RStudio PBC, Boston, MA, USA). Annual IR was calculated and expressed as the number of new cases per 100,000 person-years.

We performed two distinct comparative analyses:

1. To evaluate pandemic-related changes, we compared the IR of each disease during the post-pandemic years (2020–2023) against the pre-pandemic baseline (2019) using Pearson's chi-square tests.
2. To assess temporal trends, we conducted year-over-year comparisons of disease incidence rates using Pearson's chi-square tests.

Statistical significance was set at P < 0.05 for all analyses.

## 4 Results

This retrospective analysis examined autoimmune disease IR among children aged 0–18 between 2019 and 2023, comparing pre-pandemic (2019) with pandemic and post-pandemic periods (2020–2023). The study cohort comprised over 1.5 million children insured by Clalit Health Services (Table 1).

**Table 1:** Total number of included children per year.

Year	Number
2019	1,691,643
2020	1,483,830
2021	1,748,530
2022	1,421,099
2023	1,426,987

AID - Autoimmune Disease, AITD - Autoimmune Thyroid Diseases, GD- GDs' disease, T1D - Type 1 Diabetes DKA - Diabetic Ketoacidosis, CD - Celiac Disease, UC - Ulcerative Colitis, CrD- Crohn's Disease, AIH - Autoimmune Hepatitis, HSP - Henoch Schoenlein Purpura, JIA - Juvenile Idiopathic Arthritis, SLE - Systemic Lupus Erythematosus, ITP - Immune Thrombocytopenia Purpura.

Endocrine autoimmune manifestations showed notable temporal variations. AITD and hypothyroidism demonstrated consistent upward trends, with significant IR elevations from 2020 to 2023 compared to 2019 (P < 0.05). Significant year-over-year increases were also observed from 2021 to 2023. GD showed significant IR increases, specifically during 2022–2023, compared to 2019 baseline levels (P < 0.05). T1D exhibited significant IR increases in 2020, 2022, and 2023 compared to 2019 and 2022 compared to 2021 (P < 0.05). Notably, DKA showed a contrasting trend, with a significant initial IR increase in 2020 compared to 2019 (P < 0.05), followed by a significant decrease in 2021 compared to both years (P < 0.05), and an additional significant increase from 2022 compared to 2021 (P < 0.05) (Table 2, Table 3, Table 4).

**Table 2:** Annual IR per 100,000 person per year of autoimmune diseases each year from 2019 to 2023.

AID	2019	2020	2021	2022	2023
AITD	66.37	79.72	83.28	105.59	128.48
Hypothyroidism	40.98	39.17	41.37	51.25	58.48
GD	1.30	2.16	2.52	3.17	3.36
T1D	15.43	19.75	18.53	28.08	24.60
DKA	7.92	10.65	6.46	8.80	7.85
CD	59.50	63.12	67.30	91.49	90.83
UC	6.38	9.17	8.46	12.03	13.32
CrD	16.32	16.92	10.18	9.64	10.65
AIH	1.71	2.63	1.32	2.32	2.38
HSP	30.45	24.94	18.19	37.31	39.40
JIA	7.09	7.95	7.09	11.33	9.88
SLE	1.54	2.56	2.17	2.67	2.45
Psoriasis	87.86	98.22	81.05	112.22	122.65
Vitiligo	323.93	309.35	309.61	405.20	436.45
ITP	16.08	14.02	12.98	17.45	16.54

IR - Incidence Rate, AID - Autoimmune Disease, AITD - Autoimmune Thyroid Diseases, GD- GDs' disease, T1D - Type 1 Diabetes DKA - Diabetic Ketoacidosis, CD - Celiac Disease, UC - Ulcerative Colitis, CrD- Crohn's Disease, AIH - Autoimmune Hepatitis, HSP - Henoch Schoenlein Purpura, JIA - Juvenile Idiopathic Arthritis, SLE - Systemic Lupus Erythematosus, ITP - Immune Thrombocytopenia Purpura.

**Table 3:** Comparison of each year to pre-pandemic (2019) IR chi-square (X2) P-values results.

AID	2020	2021	2022	2023
AITD	**<0.05**	**<0.05**	**<0.05**	**<0.05**
Hypothyroidism	**<0.05**	**<0.05**	**<0.05**	**<0.05**
GD	0.09	0.26	**<0.05**	**<0.05**
T1D	**<0.05**	0.43	**<0.05**	**<0.05**
DKA	**<0.05**	**<0.05**	0.66	0.18
CD	0.20	**<0.05**	**<0.05**	**<0.05**
UC	**<0.05**	0.71	**<0.05**	**<0.05**
CrD	0.20	**<0.05**	**<0.05**	**<0.05**
AIH	0.10	**<0.05**	1.00	0.98
HSP	**<0.05**	**<0.05**	**<0.05**	**<0.05**
JIA	0.41	0.95	**<0.05**	**<0.05**
SLE	**0.05**	0.98	0.52	0.80
Psoriasis	**<0.05**	0.11	**<0.05**	**<0.05**
Vitiligo	**<0.05**	**<0.05**	**<0.05**	**<0.05**
ITP	0.15	0.60	**<0.05**	**<0.05**

IR - Incidence Rate (per 100,000 person per year), AID - Autoimmune Disease, AITD - Autoimmune Thyroid Diseases, GD- GDs' disease, T1D - Type 1 Diabetes DKA - Diabetic Ketoacidosis, CD - Celiac Disease, UC - Ulcerative Colitis, CrD- Crohn's Disease, AIH - Autoimmune Hepatitis, HSP - Henoch Schoenlein Purpura, JIA - Juvenile Idiopathic Arthritis, SLE - Systemic Lupus Erythematosus, ITP - Immune Thrombocytopenia Purpura.

**Table 4:** Comparison of each year to the preceding year IR chi-square (X2) P-values results.

AID	2021 to 2020	2022 to 2021	2023 to 2022
AITD	0.23	**<0.05**	**<0.05**
Hypothyroidism	0.34	**<0.05**	**<0.05**
GD	0.58	0.33	0.85
T1D	0.45	**<0.05**	0.08
DKA	**<0.05**	**<0.05**	0.42
CD	0.15	**<0.05**	0.87
UC	0.54	**<0.05**	0.36
CrD	**<0.05**	0.67	0.43
AIH	**<0.05**	**<0.05**	1.00
HSP	**<0.05**	**<0.05**	0.38
JIA	0.41	**<0.05**	0.26
SLE	0.55	0.43	0.80
Psoriasis	**<0.05**	**<0.05**	**<0.05**
Vitiligo	0.97	**<0.05**	**<0.05**
ITP	0.45	**<0.05**	0.59

IR - Incidence Rate (per 100,000 person per year), AID - Autoimmune Disease, AITD - Autoimmune Thyroid Diseases, GD- GDs' disease, T1D - Type 1 Diabetes DKA - Diabetic Ketoacidosis, CD - Celiac Disease, UC - Ulcerative Colitis, CrD- Crohn's Disease, AIH - Autoimmune Hepatitis, HSP - Henoch Schoenlein Purpura, JIA - Juvenile Idiopathic Arthritis, SLE - Systemic Lupus Erythematosus, ITP - Immune Thrombocytopenia Purpura.

Celiac Disease demonstrated significant IR increases from 2021 to 2023 compared to 2019, with additional elevation in 2022 versus 2021 (P < 0.05). Ulcerative Colitis showed significant IR increases in 2020, 2022, and 2023 compared to 2019 (P < 0.05), and 2022 compared to 2021 (P < 0.05), while Crohn's Disease demonstrated significant declines during 2021–2023 compared to 2019 (P < 0.05), and in 2021 compared to 2020 (P < 0.05). AIH exhibited a significant IR decrease in 2021 compared to 2019 and 2020 (P < 0.05). , followed by an increase in 2022 compared to 2021 (Table 2, Table 3, Table 4).

HSP exhibited the most consistent elevation among rheumatic manifestations, with significantly increased IR across 2020–2023 compared to 2019 (P < 0.05), including notable year-over-year increases from 2020 to 2022 (Table 2, Table 3, Table 4). JIA demonstrated significant IR elevations in 2022–2023 versus 2019 and 2022 versus 2021 (P < 0.05). SLE showed a significant increase only in 2020 compared to 2019 (P = 0.005), indicating an initial impact that subsequently stabilized (Table 2, Table 3, Table 4).

Dermatological diseases demonstrated consistent elevations, with Psoriasis and Vitiligo showing significant IR increases throughout 2020–2023 compared to 2019, including notable year-over-year increases (P < 0.05). ITP exhibited a distinct pattern, with initial non-significant decreases during 2019–2021, followed by significant increases in 2022–2023 compared to both 2019 and 2021 (P < 0.05) (Table 2, Table 3, Table 4).

## 5 Discussion

Our comprehensive study of various pediatric autoimmune diseases in a cohort of over 1.5 million children following the COVID-19 pandemic reveals distinct temporal patterns, with several conditions demonstrating significant associations with SARS-CoV-2 infection aligning with previous research on viral triggers for autoimmunity [40,42]. Multiple mechanisms underlie this relationship, including molecular mimicry between viral and self-antigens with identified cross-reactive epitopes [1,[53], [54], [55]], bystander activation of autoreactive T cells [[29], [30], [31]], cryptic antigen exposure during tissue damage [56], immune dysregulation through cytokine storms and T cell subset alterations [[32], [33], [34], [35]], and enhanced NET formation [28]. Genetic susceptibility [57] and pandemic-related environmental factors, including sedentary behavior and altered gut microbiota due to stress or dietary shifts, contributed to immune dysregulation, creating an autoimmune-prone environment. Moreover, epigenetic modifications, including altered DNA methylation and histone modification in post-COVID patients, suggest SARS-CoV-2-mediated effects on immune-regulatory gene expression [58,59]. Notably, the observed post-pandemic increase in cases may partially reflect a diagnostic “catch-up” effect from healthcare access limitations rather than solely new disease onset.

Endocrine disorders exhibited diverse patterns, with sustained AITD increases from 2020 aligning with SARS-CoV-2's documented thyroid tropism [40,[60], [61], [62], [63], [64]]. Muller et al. [60] and Speer et al. [63] documented direct thyroid involvement in COVID-19 through ACE2 receptor expression, while D'Angelo et al. [64] demonstrated post-COVID thyroid autoimmunity in pediatric populations. The delayed diagnosis surge observed between 2021 and 2023 likely reflects pandemic-related healthcare access limitations, reduced non-essential testing, and the natural infection-to-onset latency [65,66]. GD exhibited a distinct biphasic pattern with initial non-significant IR changes during 2020–2021, followed by significant increases in 2022–2023 compared to the 2019 baseline. Pathogenetic mechanisms potentially include direct thyroid damage via ACE2 receptors and indirect injury through immune dysregulation, particularly IL-6-mediated thyroiditis [63,64]. Given this temporal association and pandemic-disrupted healthcare, systematic thyroid function monitoring is warranted during acute and convalescent COVID-19 phases, particularly in high-risk patients with pre-existing thyroid conditions [63,64,67].

T1D demonstrated a noteworthy pattern; the significant increases in 2020, 2022, and 2023 align with a systematic review and meta-analysis results showing a global IR elevation from 19.73 to 32.39 per 100,000 (2019–2020) [68] and corroborating studies by Unsworth et al. [69,70] and Gottesman et al. [71]. Proposed mechanisms linking SARS-CoV-2 to T1D include pancreatic islet ACE2 receptor expression, direct β-cell damage, virus-induced inflammatory responses, and potential transient damage from viral entry [[71], [72], [73]]. However, DKA exhibited a distinct pattern, with a significant surge in 2020, followed by a substantial decrease in 2021, a resurgence in 2022, and a return to pre-pandemic levels in 2023. The initial 2020 surge featured more severe presentations, including lower pH levels, higher HbA1c, more prolonged hospitalizations, and increased PICU admissions, aligning with widespread reports [68,71,[73], [74], [75], [76]], reflecting pandemic-related healthcare barriers [74,77]. Subsequent fluctuations suggest healthcare system adaptation to pandemic conditions and evolving diabetes management strategies or changes in patient behavior and healthcare access over time [78]. Notably, the DKA pattern, which includes previously diagnosed diabetes patients, was likely influenced by reduced access to insulin, continuous glucose monitoring sensors, and medical consultations during the pandemic outbreak. This divergence between T1D and DKA trends highlights the complex interplay between pandemic conditions, healthcare accessibility, and diabetes management [74,[77], [78], [79]].

Gastrointestinal autoimmune diseases demonstrated complex trends, as observed in recent publications [[80], [81], [82]], reflecting the complexity of gut-immune interactions with viral infections [83]. CD showed consistent increases from 2019 through 2022 before plateauing in 2023, corresponding with evidence of SARS-CoV-2's impact on intestinal immunity and microbiota composition [[84], [85], [86], [87]]. The marked increase between 2021 and 2022 partially reflects the 2020 ESPGHAN guidelines implementation, which broadened diagnostic criteria allowing serological diagnosis without biopsies in asymptomatic children [88]. The IR plateaued in 2023, suggesting a stabilization following the rapid increase in the preceding years. UC displayed significant elevations in 2022–2023 compared to 2019, potentially reflecting COVID-19-associated immune dysregulation [80,89,90]. Crohn's Disease showed a distinctive decrease during 2020–2021, coinciding with social isolation periods, contrasting with previous Israeli pediatric trends, and suggesting distinct pathogenic factors from UC [80,81]. AIH exhibited a characteristic pattern of decreased IR in 2021, followed by a significant surge in 2022, likely reflecting pandemic-related healthcare access restrictions and subsequent compensatory diagnoses. However, its low absolute incidence warrants cautious interpretation [83].

Among rheumatic diseases, HSP demonstrated consistent, statistically significant increases throughout 2020–2023 compared to 2019, aligning with documented post-SARS-CoV-2 HSP cases [91,92]. This association is supported by the virus's direct effect on vascular endothelium through ACE2 receptor binding [93,94], triggering endothelial dysfunction, complement activation, and immune complex deposition — the hallmark features of HSP vasculitis as detailed in comprehensive reviews [[93], [94], [95], [96], [97], [98], [99]]. JIA exhibited a distinctive delayed pattern with significant increases in 2022–2023, reflecting potential latency between viral exposure and autoimmune manifestation. A comprehensive review by Rojas et al. [100] detailed how molecular mimicry between SARS-CoV-2 peptides and synovial antigens, combined with virus-induced cytokine dysregulation, could trigger JIA in genetically susceptible individuals [42,[100], [101], [102]]. SLE showed increased IR between 2020–2019, potentially linked to shared pathogenic mechanisms with COVID-19, including interferon pathway activation and NET formation [24,28]. This overall increase in pediatric rheumatic disease IR is likely multifactorial, involving post-viral immune dysregulation, lifestyle changes, healthcare access issues, and genetic predisposition [[96], [97], [98], [99]].

Dermatological autoimmune conditions exhibited some of the most consistent trends in our study, with both Psoriasis and Vitiligo showing sustained elevation in IR. These trends align with reported cases of new-onset and exacerbated disease following SARS-CoV-2 infection, likely due to viral skin tropism, melanocyte impact, and systemic inflammatory responses in both adults and children, likely [103]. While pediatric post-COVID-19 psoriasis literature remains limited, documented cases suggest molecular mimicry, bystander killing, and epitope spreading as potential mechanisms for both new-onset diseases and exacerbation of existing conditions [104,105]. Similarly, post-infection and post-vaccination Vitiligo cases indicate possible immune-mediated melanocyte targeting through viral antigen cross-reactivity [[106], [107], [108], [109]].

Hematological manifestations, particularly ITP, exhibited a distinctive biphasic pattern with an initial decrease followed by significant IR increases, reflecting early social distancing effects and subsequent SARS-CoV-2-triggered autoimmune responses [110]. COVID-19-associated pediatric ITP cases typically presented with thrombocytopenia but minimal bleeding complications, with a median 17-day time to diagnosis [111]. Proposed pathogenic mechanisms include viral-induced endothelial injury and antibody cross-reactivity between viral glycoproteins and platelet surface integrins, with 31 % of cases presenting without bleeding and rare occurrence of severe hemorrhagic complications [112,113].

Our study has several limitations that warrant consideration. First, the observed changes in IR may be influenced by factors other than COVID-19, including altered healthcare-seeking behaviors, lockdown-related diagnostic delays, and vaccination program implementation (2021–2022). Without individual vaccination data and timing relative to disease onset, we cannot distinguish between infection and vaccination-associated autoimmune manifestations. Second, the retrospective nature of our study limits the establishment of a causal relationship between COVID-19, vaccination, and autoimmune disease onset. Despite these limitations, our study possesses several notable strengths. The primary strength lies in its extensive cohort size of over 1.5 million children, making it one of the largest studies of its kind. The study's comprehensive scope also encompasses multiple autoimmune conditions across various medical specialties, providing a broad perspective on autoimmune manifestations. Furthermore, the three-year follow-up period allows for robust temporal analysis of disease patterns and trends throughout different phases of the pandemic and vaccination periods.

Future research priorities should encompass long-term follow-up studies examining trend persistence and disease prognosis, investigating potential genetic and environmental factors predisposing individuals to post-COVID autoimmune conditions, and comprehensive analyses incorporating vaccination and confirmed infection status histories. Additionally, mechanistic studies utilizing animal models and in vitro experiments are needed to elucidate SARS-CoV-2's role in triggering autoimmunity and evaluate vaccination's impact on disease incidence, given reports of autoimmune phenomena following vaccination [55,114]. This multifaceted approach will enhance understanding of the pandemic's long-term implications for pediatric autoimmune diseases and inform future prevention and treatment strategies.

## 6 Conclusion

In conclusion, our big data analysis reveals significant changes in the IR of various pediatric autoimmune diseases following the COVID-19 pandemic, with distinct patterns and notable increases observed across multiple conditions. Although some changes may reflect pandemic-related healthcare disruptions, the persistence and diversity of these changes suggest a complex relationship between SARS-CoV-2 infection and autoimmune dysregulation in pediatric populations. These findings emphasize the critical importance of enhanced clinical surveillance, systematic long-term follow-up of affected children, and continued research into the pathophysiological mechanisms to develop targeted prevention and treatment strategies for post-COVID autoimmune manifestations.

## CRediT authorship contribution statement

**Rim Kasem Ali Sliman:** Writing – review & editing, Writing – original draft, Visualization, Validation, Supervision, Project administration, Methodology, Investigation, Formal analysis, Data curation, Conceptualization. **Hilla Cohen:** Writing – review & editing, Project administration, Methodology, Formal analysis, Data curation. **Shereen Shehadeh:** Writing – review & editing, Writing – original draft. **Reut Batcir:** Writing – review & editing, Writing – original draft. **Yigal Elenberg Alter:** Writing – review & editing, Writing – original draft. **Keren Cohen:** Writing – review & editing, Writing – original draft. **Ilana Koren:** Writing – review & editing, Writing – original draft. **Inbal Halabi:** Writing – review & editing, Writing – original draft. **Hussein Sliman:** Writing – review & editing, Writing – original draft. **Mohamad Hamad Saied:** Writing – review & editing, Writing – original draft, Visualization, Supervision, Project administration, Methodology, Conceptualization.

## Funding

The authors declare that no funds, grants, or other support were received during the preparation of this manuscript.

## Declaration of competing interest

The authors declare that they have no known competing financial interests or personal relationships that could have appeared to influence the work reported in this paper.

## Data Availability

Data will be made available on request.

## References

[1] Ehrenfeld M., Tincani A., Andreoli L. (2020). Covid-19 and autoimmunity. Autoimmun. Rev..

[2] Gutierrez-Arcelus M., Rich S.S., Raychaudhuri S., Raychaudhuri S. (2016). Autoimmune diseases - connecting risk alleles with molecular traits of the immune system. Nat. Rev. Genet..

[3] Levy M., Kolodziejczyk A.A., Thaiss C.A., Elinav E. (2017). Dysbiosis and the immune system. Nat. Rev. Immunol..

[4] Ercolini A.M., Miller S.D., Miller S.D. (2009). The role of infections in autoimmune disease. Clin. Exp. Immunol..

[5] Vojdani A., Vojdani A. (2014). A potential link between environmental triggers and autoimmunity. Autoimmune Dis..

[6] Ngo S.T., Steyn F.J., McCombe P.A., McCombe P.A. (2014). Gender differences in autoimmune disease. Front. Neuroendocrinol..

[7] Theofilopoulos A.N., Kono D.H., Baccala R., Baccala R. (2017). The multiple pathways to autoimmunity. Nat. Immunol..

[8] Winchester N., Calabrese C., Calabrese L.H. (2021). The intersection of COVID-19 and autoimmunity: what is our current understanding?. Pathog. Immun..

[9] Almishal R.O., Yassine H.M. (2019).

[10] Gracia-Ramos A.E., Martin-Nares E., Hernández-Molina G., Hernández-Molina G. (2021). New onset of autoimmune diseases following COVID-19 diagnosis. Cells.

[11] Kamen D.L., Kamen D.L. (2014). Environmental influences on systemic lupus erythematosus expression. Rheum. Dis. Clin. N. Am..

[12] Costenbader K.H., Karlson E.W., Karlson E.W. (2006). Epstein-Barr virus and rheumatoid arthritis: is there a link?. Arthritis Res. Ther..

[13] Lidar M., Lipschitz N., Langevitz P., Shoenfeld Y. (2009). The infectious etiology of vasculitis. Autoimmunity.

[14] Tomer Y., Davies T.F., Davies T.F. (1993). Infection, thyroid disease, and autoimmunity. Endocr. Rev..

[15] Kerr J.R., Kerr J.R. (2016). The role of parvovirus B19 in the pathogenesis of autoimmunity and autoimmune disease. J. Clin. Pathol..

[16] Ascherio A., Munger K.L., Munger K.L. (2015). EBV and autoimmunity. Curr. Top. Microbiol. Immunol..

[17] Halenius A., Hengel H., Hengel H. (2014). Human cytomegalovirus and autoimmune disease. BioMed Res. Int..

[18] Cacoub P., Comarmond C., Comarmond C., Comarmond C. (2017). New insights into HCV-related rheumatologic disorders: a review. J. Adv. Res..

[19] Ferri C., Ramos-Casals M., Zignego A.L. (2016). International diagnostic guidelines for patients with HCV-related extrahepatic manifestations. A multidisciplinary expert statement. Autoimmun. Rev..

[20] Hyöty H., Hyöty H. (2016). Viruses in type 1 diabetes. Pediatr. Diabetes.

[21] Dotan A., Muller S., Kanduc D., David P., Halpert G., Shoenfeld Y. (2021). The SARS-CoV-2 as an instrumental trigger of autoimmunity. Autoimmun. Rev..

[22] Tough D.F., Borrow P., Sprent J., Sprent J. (1996). Induction of bystander T cell proliferation by viruses and type I interferon in vivo. Science.

[23] Münz C., Lünemann J.D., Getts M.T., Miller S.D. (2009). Antiviral immune responses: triggers of or triggered by autoimmunity?. Nat. Rev. Immunol..

[24] Bastard P., Rosen L.B., Zhang Q. (2020). Autoantibodies against type I IFNs in patients with life-threatening COVID-19. Science.

[25] Fujinami R.S., Von Herrath M.G., Christen U., Whitton J.L. (2006). Molecular mimicry, bystander activation, or viral persistence: infections and autoimmune disease. Clin. Microbiol. Rev..

[26] Hussein H.M., Rahal E.A. (2019). The role of viral infections in the development of autoimmune diseases. Crit. Rev. Microbiol..

[27] Scherer M.T., Ignatowicz L., Winslow G.M., Kappler J.W., Marrack P. (1993). Superantigens: bacterial and viral proteins that manipulate the immune system. Annu. Rev. Cell Biol..

[28] Zuo Y., Yalavarthi S., Shi H. (2020). Neutrophil extracellular traps in COVID-19. JCI Insight.

[29] Fujinami R.S., von Herrath M.G., Christen U., Whitton J.L. (2006). Molecular mimicry, bystander activation, or viral persistence: infections and autoimmune disease. Clin. Microbiol. Rev..

[30] Rodríguez Y., Novelli L., Rojas M. (2020). Autoinflammatory and autoimmune conditions at the crossroad of COVID-19. J. Autoimmun..

[31] Galeotti C., Bayry J., Bayry J. (2020). Autoimmune and inflammatory diseases following COVID-19. Nat. Rev. Rheumatol..

[32] Merad M., Martin J.C., Martin J.C. (2020). Pathological inflammation in patients with COVID-19: a key role for monocytes and macrophages. Nat. Rev. Immunol..

[33] Lee J.S., Park S., Jeong H.W. (2020). Immunophenotyping of COVID-19 and influenza highlights the role of type I interferons in development of severe COVID-19. Sci. Immunol..

[34] Liu Y., Sawalha A.H., Lu Q., Lu Q. (2021). COVID-19 and autoimmune diseases. Curr. Opin. Rheumatol..

[35] Rizk J.G., Kalantar-Zadeh K., Mehra M.R., Lavie C.J., Rizk Y., Forthal D.N. (2020). Pharmaco-immunomodulatory therapy in COVID-19. Drugs.

[36] El-Hor N., Adams M. (2021). Pediatric rheumatologic effects of COVID-19. Pediatr. Clin. North Am..

[37] Freites Nuñez D.D., Leon L., Mucientes A. (2020). Risk factors for hospital admissions related to COVID-19 in patients with autoimmune inflammatory rheumatic diseases. Ann. Rheum. Dis..

[38] City N.Y., Appendix S. (2020).

[39] Batu E.D., Sener S., Ozen S. (2022). COVID-19 associated pediatric vasculitis: a systematic review and detailed analysis of the pathogenesis. Semin. Arthritis Rheum..

[40] Caso F., Costa L., Ruscitti P. (2020). Could Sars-coronavirus-2 trigger autoimmune and/or autoinflammatory mechanisms in genetically predisposed subjects?. Autoimmun. Rev..

[41] Mohkhedkar M., Venigalla S.S.K., Janakiraman V. (2021). Untangling COVID-19 and autoimmunity: identification of plausible targets suggests multi organ involvement. Mol. Immunol..

[42] Vojdani A., Kharrazian D., Kharrazian D. (2020). Potential antigenic cross-reactivity between SARS-CoV-2 and human tissue with a possible link to an increase in autoimmune diseases. Clin. Immunol..

[43] Tutal E., Ozaras R., Leblebicioglu H. (2022). Systematic review of COVID-19 and autoimmune thyroiditis. Trav. Med. Infect. Dis..

[44] Novelli L., Motta F., De Santis M., Ansari A.A., Gershwin M.E., Selmi C. (2021). The JANUS of chronic inflammatory and autoimmune diseases onset during COVID-19 – a systematic review of the literature. J. Autoimmun..

[45] Zulfiqar A.A., Lorenzo-Villalba N., Hassler P., Andrès E. (2020). Immune thrombocytopenic purpura in a patient with Covid-19. N. Engl. J. Med..

[46] Toscano G., Palmerini F., Ravaglia S. (2020). Guillain-Barré syndrome associated with SARS-CoV-2. N. Engl. J. Med..

[47] Becker R.C., Becker R.C. (2020). COVID-19-associated vasculitis and vasculopathy. J. Thromb. Thrombolysis.

[48] Wood H., Jones J.R., Hui K. (2020). Secondary HLH is uncommon in severe COVID-19. Br. J. Haematol..

[49] Verdoni L., Mazza A., Gervasoni A. (2020). An outbreak of severe Kawasaki-like disease at the Italian epicentre of the SARS-CoV-2 epidemic: an observational cohort study. Lancet.

[50] Jacobs J., Eichbaum Q., Eichbaum Q. (2021). COVID-19 associated with severe autoimmune hemolytic anemia. Transfusion (Paris).

[51] Muller I., Cannavaro D., Dazzi D. (2020). SARS-CoV-2-related atypical thyroiditis. Lancet Diabetes Endocrinol..

[52] Parsons T., Banks S., Bae C., Gelber J., Alahmadi H., Tichauer M. (2020). COVID-19-associated acute disseminated encephalomyelitis (ADEM). J. Neurol..

[53] Angileri F., Legare S., Marino Gammazza A., Conway de Macario E., Jl Macario A., Cappello F. (2020). Molecular mimicry may explain multi-organ damage in COVID-19. Autoimmun. Rev..

[54] Lyons-Weiler J., Lyons-Weiler J. (2020). Pathogenic priming likely contributes to serious and critical illness and mortality in COVID-19 via autoimmunity. J. Transl. Autoimmun..

[55] Vojdani A., Kharrazian D., Kharrazian D. (2020). Potential antigenic cross-reactivity between SARS-CoV-2 and human tissue with a possible link to an increase in autoimmune diseases. Clin. Immunol..

[56] Caso F., Costa L., Ruscitti P. (2020). Could Sars-coronavirus-2 trigger autoimmune and/or autoinflammatory mechanisms in genetically predisposed subjects?. Autoimmun. Rev..

[57] Beyerstedt S., Casaro E.B., Rangel É.B., Rangel É.B. (2021). COVID-19: angiotensin-converting enzyme 2 (ACE2) expression and tissue susceptibility to SARS-CoV-2 infection. Eur. J. Clin. Microbiol. Infect. Dis..

[58] Lim S.H., Ju H.J., Han J.H. (2023). Autoimmune and autoinflammatory connective tissue disorders following COVID-19. JAMA Netw. Open.

[59] Roseti L., Grigolo B., Grigolo B. (2022). COVID-19 and rheumatic diseases: a mini-review. Front. Med..

[60] Muller I., Cannavaro D., Dazzi D. (2020). SARS-CoV-2-related atypical thyroiditis. Lancet Diabetes Endocrinol..

[61] Scappaticcio L., Pitoia F., Esposito K., Piccardo A., Trimboli P. (2021). Impact of COVID-19 on the thyroid gland: an update. Rev. Endocr. Metab. Disord..

[62] Rodríguez Y., Novelli L., Rojas M. (2020). Autoinflammatory and autoimmune conditions at the crossroad of COVID-19. J. Autoimmun..

[63] Speer G., Somogyi P., Somogyi P., Speer G. (2021). Thyroid complications of SARS and coronavirus disease 2019 (COVID-19). Endocr. J..

[64] d'Aniello F., Amodeo M.E., Grossi A., Ubertini G. (2024). Thyroiditis and COVID-19: focus on pediatric age. A narrative review. J. Endocrinol. Investig..

[65] Goldman S., Pinhas-Hamiel O., Weinberg A. (2022). Alarming increase in ketoacidosis in children and adolescents with newly diagnosed type 1 diabetes during the first wave of the COVID-19 pandemic in Israel. Pediatr. Diabetes.

[66] Yazdanpanah N., Rezaei N., Rezaei N. (2022). Autoimmune complications of COVID-19. J. Med. Virol..

[67] Tutal E., Ozaras R., Leblebicioglu H., Leblebicioglu H. (2022). Systematic review of COVID-19 and autoimmune thyroiditis. Trav. Med. Infect. Dis..

[68] Mirnasuri S., Rahmati M., Keshvari M., Mirnasuri S. (2022). The global impact of COVID‐19 pandemic on the incidence of pediatric new‐onset type 1 diabetes and ketoacidosis: a systematic review and meta‐analysis. J. Med. Virol..

[69] Rubino F., Amiel S.A., Zimmet P. (2020). New-onset diabetes in Covid-19. N. Engl. J. Med..

[70] Unsworth R., Wallace S., Oliver N.S. (2020). New-onset type 1 diabetes in children during COVID-19: multicenter regional findings in the U.K. Diabetes Care.

[71] Gottesman B.L., Yu J., Tanaka C., Longhurst C.A., Kim J.J. (2022). Incidence of new-onset type 1 diabetes among US children during the COVID-19 global pandemic. JAMA Pediatr..

[72] Johnson D., Mannheim J., Johnson D. (2024). COVID-19 and diabetes: an epidemiologic overview. Pediatr. Ann..

[73] Montefusco L., Bolla A.M., Fiorina P., Fiorina P. (2022). Should we expect a wave of type 1 diabetes following SARS-CoV-2 pandemic?. Diabetes Metab. Res. Rev..

[74] Goldman S., Pinhas-Hamiel O., Weinberg A. (2022). Alarming increase in ketoacidosis in children and adolescents with newly diagnosed type 1 diabetes during the first wave of the COVID-19 pandemic in Israel. Pediatr. Diabetes.

[75] Ponmani C., Sakka S., Wickramarachchi C. (2021). 506 Characteristics of new-onset paediatric Type 1 diabetes in the COVID-19 pandemic – a multicentre perspective. Abstracts.

[76] Kamrath C., Mönkemöller K., Biester T. (2020). Ketoacidosis in children and adolescents with newly diagnosed type 1 diabetes during the COVID-19 pandemic in Germany. JAMA.

[77] Klatman E.L., Besançon S., Bahendeka S., Mayige M., Ogle G.D. (2020). COVID-19 and type 1 diabetes: challenges and actions. Diabetes Res. Clin. Pract..

[78] Vlad A., Serban V., Timar R. (2021). Increased incidence of type 1 diabetes during the COVID-19 pandemic in Romanian children. Medicina (Kaunas).

[79] Odeh R., Gharaibeh L., Daher A., Kussad S., Alassaf A. (2022). Caring for a child with type 1 diabetes during COVID-19 lockdown in a developing country : challenges and parents ’ perspectives on the use of telemedicine. Diabetes Res. Clin. Pract..

[80] Kuenzig M.E., Fung S.G., Marderfeld L. (2022). Twenty-first century trends in the global epidemiology of pediatric-onset inflammatory Bowel disease: systematic review. Gastroenterology.

[81] Stulman M.Y., Asayag N., Focht G. (2021). Epidemiology of inflammatory Bowel diseases in Israel: a nationwide epi-Israeli IBD research nucleus study. Inflamm. Bowel Dis..

[82] Conrad N., Misra S., Verbakel J.Y. (2023). Incidence, prevalence, and co-occurrence of autoimmune disorders over time and by age, sex, and socioeconomic status: a population-based cohort study of 22 million individuals in the UK. Lancet.

[83] Vlad A., Serban V., Timar R. (2021). Increased incidence of type 1 diabetes during the COVID-19 pandemic in Romanian children. Medicina (Kaunas).

[84] Lui G.C.Y., Yeoh Y.K., Zuo T., Lui G.C.Y. (2021). Gut microbiota composition reflects disease severity and dysfunctional immune responses in patients with COVID-19. Gut.

[85] Zuo T., Zhang F., Lui G.C.Y. (2020). Alterations in gut microbiota of patients with COVID-19 during time of hospitalization. Gastroenterology.

[86] Lamers MM, Beumer J, van der Vaart J, Knoops K, Puschhof J, Breugem TI, Ravelli RB, Paul van Schayck J, Mykytyn AZ, Duimel HQ, van Donselaar E (2020 Jul 3). SARS-CoV-2 productively infects human gut enterocytes. Science.

[87] Garg M., Royce S.G., Tikellis C. (2020). Imbalance of the renin-angiotensin system may contribute to inflammation and fibrosis in IBD: a novel therapeutic target?. Gut.

[88] Husby S., Koletzko S., Korponay-Szabó I. (2020). European society paediatric Gastroenterology, Hepatology and nutrition guidelines for diagnosing coeliac disease 2020. J. Pediatr. Gastroenterol. Nutr..

[89] Qin C., Zhou L., Hu Z. (2020). Dysregulation of immune response in patients with coronavirus 2019 (COVID-19) in Wuhan, China. Clin. Infect. Dis..

[90] Mazzoni A., Salvati L., Maggi L., Annunziato F., Cosmi L. (2021). Hallmarks of immune response in COVID-19: exploring dysregulation and exhaustion. Semin. Immunol..

[91] Diorio C., Henrickson S.E., Vella L.A. (2020). Multisystem inflammatory syndrome in children and COVID-19 are distinct presentations of SARS-CoV-2. J. Clin. Investig..

[92] Java A., Apicelli A.J., Liszewski M.K. (2020). The complement system in COVID-19: friend and foe?. JCI Insight.

[93] Toubiana J., Poirault C., Corsia A. (2020). Kawasaki-like multisystem inflammatory syndrome in children during the covid-19 pandemic in Paris, France: prospective observational study. BMJ.

[94] Rowley A.H., Rowley A.H. (2020). Understanding SARS-CoV-2-related multisystem inflammatory syndrome in children. Nat. Rev. Immunol..

[95] Mehta P., McAuley D.F., Brown M. (2020). COVID-19: consider cytokine storm syndromes and immunosuppression. Lancet.

[96] Ruscitti P., Caso F., Costa L., Ruscitti P. (2020). Could Sars-coronavirus-2 trigger autoimmune and/or autoinflammatory mechanisms in genetically predisposed subjects?. Autoimmun. Rev..

[97] Diao B., Wang C., Wang R. (2021). Human kidney is a target for novel severe acute respiratory syndrome coronavirus 2 infection. Nat. Commun..

[98] Roncati L., Ligabue G., Fabbiani L. (2020). Type 3 hypersensitivity in COVID-19 vasculitis. Clin. Immunol..

[99] Varga Z., Flammer A.J., Steiger P. (2020). Endothelial cell infection and endotheliitis in COVID-19. Lancet.

[100] Rojas M., Restrepo-Jiménez P., Monsalve D.M. (2018). Molecular mimicry and autoimmunity. J. Autoimmun..

[101] Galeotti C., Bayry J., Bayry J. (2020). Autoimmune and inflammatory diseases following COVID-19. Nat. Rev. Rheumatol..

[102] Smatti M.K., Cyprian F.S., Nasrallah G.K., Al Thani A.A., Almishal R.O., Yassine H.M. (2019). Viruses and autoimmunity: a review on the potential interaction and molecular mechanisms. Viruses.

[103] Criado P.R., Abdalla B.M.Z., de Assis I.C., van Blarcum de Graaff Mello C., Caputo G.C., Vieira I.C. (2020). Are the cutaneous manifestations during or due to SARS-CoV-2 infection/COVID-19 frequent or not? Revision of possible pathophysiologic mechanisms. Inflamm. Res..

[104] Qureshi N.K., Bansal S.K., Bansal S.K., Kasapcopur O. (2021). Autoimmune thyroid disease and Psoriasis vulgaris after COVID-19 in a male teenager. Case Rep. Pediatr..

[105] Thakur V., Ratho R.K., Kumar P. (2021). Multi-organ involvement in COVID-19: beyond pulmonary manifestations. J. Clin. Med..

[106] Beltrán S., Rodríguez Y., Rojas M., Beltrán S. (2022). Autoimmune and autoinflammatory conditions after COVID-19 vaccination. New case reports and updated literature review. J. Autoimmun..

[107] Viglizzo G., Herzum A., Gariazzo L., Olcese C., Occella C. (2023). Childhood vitiligo developing after COVID-19 infection. Dermatol. Rep..

[108] Abrar E., Bukhari M. (2022). New-onset of vitiligo in a child following COVID-19 vaccination. JAAD Case Rep..

[109] Militello M., Muacevic A., Adler J.R., Militello M. (2022). Vitiligo possibly triggered by COVID-19 vaccination. Cureus.

[110] Bhattacharjee S., Banerjee M., Banerjee M. (2020). Immune thrombocytopenia secondary to COVID-19: a systematic review. SN Compr. Clin. Med..

[111] Kitagawa I., Ono R., Kitagawa I. (2024). SARS-CoV-2 infection-induced immune thrombocytopenia: a systematic review of current reports. Ann. Hematol..

[112] Mahmoudian-Sani M.R., Bahadoram M., Saeedi-Boroujeni A., Mahmoudian-Sani M.R. (2022). COVID-19-induced immune thrombocytopenic purpura; Immunopathogenesis and clinical implications. Infez Med..

[113] Yazdanpanah N., Rezaei N., Rezaei N. (2022). Autoimmune complications of COVID-19. J. Med. Virol..

[114] Watad A., De Marco G., Mahajna H. (2021). Immune-mediated disease flares or new-onset disease in 27 subjects following mRNA/DNA SARS-CoV-2 vaccination. Vaccines (Basel).

